# Genetic characterization of *psp *encoding the DING protein in *Pseudomonas fluorescens *SBW25

**DOI:** 10.1186/1471-2180-7-114

**Published:** 2007-12-18

**Authors:** Xue-Xian Zhang, Ken Scott, Rebecca Meffin, Paul B Rainey

**Affiliations:** 1 School of Biological Sciences, University of Auckland, Private Bag 92019, Auckland, New Zealand; 2 Institute of Molecular Biosciences and NZ Institute for Advanced Study, Massey University, Private Bag 102904, Auckland, New Zealand

## Abstract

**Background:**

DING proteins constitute a conserved and broadly distributed set of proteins found in bacteria, fungi, plants and animals (including humans). Characterization of DING proteins from animal and plant tissues indicated ligand-binding ability suggesting a role for DING proteins in cell signaling and biomineralization. Surprisingly, the genes encoding DING proteins in eukaryotes have not been identified in the eukaryotic genome or EST databases. Recent discovery of a DING homologue (named Psp here) in the genome of *Pseudomonas fluorescens *SBW25 provided a unique opportunity to investigate the physiological roles of DING proteins. *P. fluorescens *SBW25 is a model bacterium that can efficiently colonize plant surfaces and enhance plant health. In this report we genetically characterize Psp with a focus on conditions under which *psp *is expressed and the protein exported.

**Results:**

Psp is closely related to the periplasmic P_i _binding component of the ABC-type phosphate transporter system (Pst). *psp *is flanked by a gene cluster predicted to function as a type II protein secretion system (Hxc). Deletion analysis combined with chromosomally integrated '*lacZ *fusions showed that both *psp *and *pstC *are induced by P_i _limitation and that *pstC *is required for competitive growth of the bacterium in P_i _limited medium. *hxcR *is not regulated by P_i _limitation. Psp was detected (using anti-DING serum) in the supernatant of wild-type culture but was greatly reduced in the supernatant of an isogenic strain carrying an *hxcR *mutation (Δ*hxcR*). A promoter fusion between *hxcR *and a promoterless copy of a gene ('*dapB*) essential for growth in the plant environment showed that expression of *hxcR *is elevated during colonization of sugar beet seedlings. A similar analysis of *psp *showed that it is not induced in the plant environment.

**Conclusion:**

Psp gene is expressed under conditions of P_i _limitation. It is an exoprotein secreted mainly via the Hxc type II secretion system, whose expression is elevated on plant surfaces. We propose that Psp is involved in extracellular scavenging of phosphates, which are subsequently taken up by the cell-bound Pst transport system.

## Background

DING proteins, named for the highly-conserved N-terminal sequence (DINGGG-), were initially described as proteins from animal and plant tissues with molecular weights about 38–40 kDa. The first DING protein was identified from human rheumatoid arthritis (RA) synovial fluid as a lymphocyte stimulatory protein [1]. Since then human DING proteins have been identified independently from urine and kidney stones as crystal adhesion inhibitors [2]; in skin fibroblasts and cervical carcinoma cells using hirudin-agarose affinity columns [3]; and in breast cells by their high affinity for genistein, an estrogen analog [4]. In these latter two cases, extracellular DING protein is linked to cell growth promotion by an autocrine or paracrine mechanism [3,4]. DING proteins have also been reported from other animals, e.g., turkey (as a lipid-free polysaccharide-binding protein) and rat (as a cotinine receptor). DING proteins from several plant and fungal species are characterized by short N-terminal sequences and one has been shown capable of binding a germin-like protein in tobacco [5]. Together these studies suggested that DING proteins are widespread in eukaryotes and play important roles in cell signaling and biomineralization.

Although DING proteins have been frequently isolated or identified from eukaryotes on the basis of ligand-binding properties, no complete gene or protein sequences have been found in either the current eukaryotic genomes or EST databases. Recently, Morales *et al*. [6] reported the first complete amino acid sequence of a DING protein isolated from the human plasma high-density lipoprotein fraction, though no corresponding gene sequence has been identified. This DING protein was structurally characterized to be a phosphate binding protein, which is functionally related to solute binding proteins of the Pst systems, the bacterial ABC (ATP-binding cassette) transporters specific for phosphate [6]. The Pst system is comprised of four components [7]: a periplasmic phosphate binding protein (PstS), two integral membrane domains (PstC and PstA) and a cytoplasmic ATPase (PstB).

Low-level homology between eukaryotic DING proteins and bacterial phosphate-binding proteins has been known for some time [5]. More recently, proteins with a greater degree of homology have been predicted in the sequenced genomes of *Pseudomonas *[8], including *P. fluorescens *SBW25 [9]. *P. fluorescens *SBW25 is a Gram-negative plant growth-promoting rhizobacterium (PGPR) representative of a group of fluorescent pseudomonads isolated from the field-grown sugar beets at the University of Oxford farm, Wytham, Oxford, in 1989 [10]. The DING homologue of *P. fluorescens *SBW25, named Psp here for phosphate scavenging protein, shows 65–75% identity with the partial eukaryotic DING protein sequences and contains the highly conserved phosphate-binding site [9]. However, prediction of flanking genes in the *psp *locus was inaccurate when the analysis was based on raw SBW25 shotgun genome sequence data available at that time [9]. The coding region of *psp *of *P. fluorescens *SBW25 was previously cloned and expressed in *Escherichia coli *and subsequent phosphate binding assays showed that the expressed SBW25 DING protein is able to bind inorganic phosphate (P_i_)* in vitro *[9]. Secondary and tertiary structure prediction and determination indicated a PstS-like, "Venus flytrap" structure [9,11,12]. A similar structure has been found for the human plasma phosphate binding protein [6]. Previous study also showed that the DING protein was not detectable in SBW25 cells grown in Luria-Bertani (LB) medium [9], suggesting that *psp *was not constitutively expressed.

In this report we describe the genetic characterization of *psp *(*pflu2427*) encoding the DING protein of *P. fluorescens *SBW25. We focus on the conditions under which *psp *is expressed and its role for bacterial growth in laboratory media and in the plant environment. Our work begins with the complete but un-annotated whole genome sequence of SBW25. Based initially on this sequence, we perform an up-to-date comparison of gene sequences of the phosphate binding proteins in SBW25 and other genome-sequenced *Pseudomonas*. We examine the functionalities of *psp*, together with the genes located upstream of *psp *(*hxcRS*) and genes in the *pst *locus, using a combination of site-directed mutagenesis and chromosomally integrated '*lacZ *fusions. The role of the DING protein in bacterial phosphate metabolism and plant colonization is discussed.

## Results

### Genomic analyses of putative phosphate-binding proteins in *Pseudomonas*

The genome of *P. fluorescens *SBW25 harbors two putative phosphate-binding proteins, Psp (Pflu2427) and PstS (Pflu3318), which show 38% amino acid sequence similarity to each other. Both Psp and PstS possess the highly conserved phosphate-binding sites [5]. However, PstS is 52 amino acids shorter than Psp and it does not contain the N-terminal DINGGG residue.

**Figure 1:**
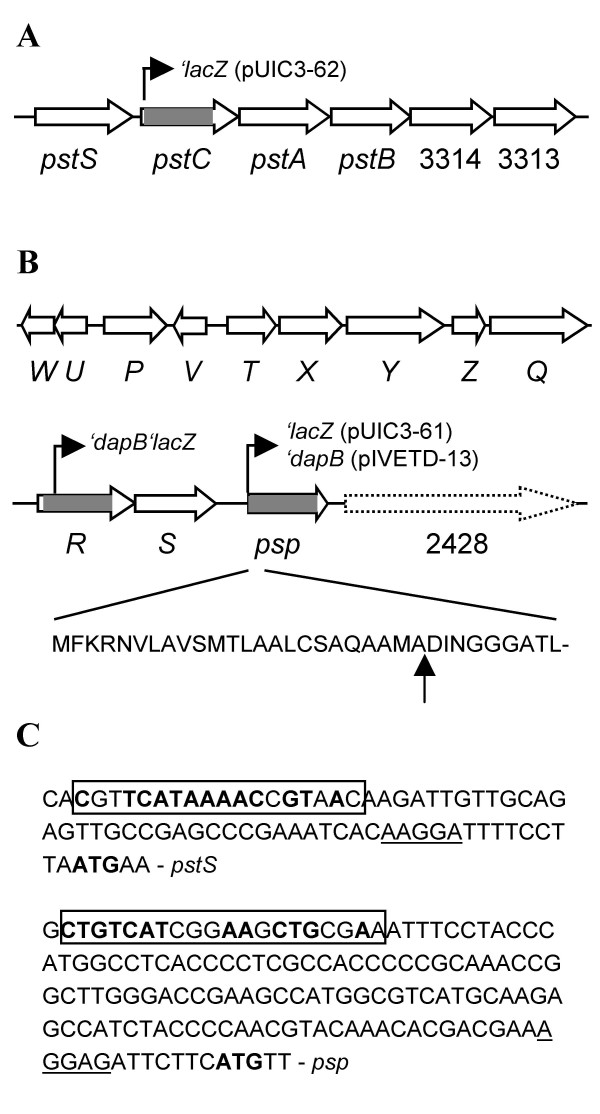
**Genetic organization of the *pst *(A) and *psp *(B) loci in *P. fluorescens *SBW25**. Regions deleted in mutants PBR827 (Δ*pstC *or *Δpflu3317*), PBR828 (Δ*hxcR *or Δ*pflu2424*) and PBR826 (Δ*psp *or Δ*pflu2427*) are marked by grey. Positions of the promoterless '*lacZ *and '*dapB *fusions are shown by arrowed lines. The predicted signal peptide cleavage site is indicated by vertical arrow. ORF Pflu2428 is not drawn to scale and shown by discontinued arrow bar. **(C) **Promoter regions of *pst *and *psp *operons. The putative translational start and ribosome-binding sites are indicated by bold type and underlined letters, respectively. The predicted Pho box sequences are boxed and nucleotides identified in the *E. coli *Pho box consensus [CTGTCATA(AT)A(TA)CTGT(CA)A(CT)] [7] are highlighted by bold type.

*pstS *is the first of six genes which are organized in the same orientation, suggesting that they are co-transcribed as an operon (Figure 1A). Genes encoding the other three components of the Pst system (PstCAB) are located downstream of *pstS*. The putative *pst *genes are organized by a gene order typical for ABC transporters involved in substrate uptake: the periplasmic binding protein (PstS) first and then the membrane-spanning domains (PstC and PstA) and the ATP-binding cassette (PstB).

*psp *is located 1035 Kb away from *pstS*. ORF *pflu2428*, downstream of *psp*, encodes a predicted protein of 4083 amino acids, which contains a haemagglutination activity domain (Figure 1B). This domain is typical of haemagglutinins, which are involved in bacterial adhesion to host cells [13]. Immediately upstream of *psp *is a cluster of 11 genes that are predicted to encode an alternative type II secretion system (Hxc) [14]. Type II-dependent exoproteins have an N-terminal signal peptide that is cleaved off during translocation to the periplasm by the Sec or Tat export system [15]. The folded or mature substrate protein is subsequently transported across the outer membrane by the type II secretion apparatus. The discovery of *hxc *flanking *psp *led to a signal peptide analysis of the deduced amino acid sequence of *psp *by using the SignalP 3.0 Server [16]. Results showed that Psp does contain a signal peptide and the predicted cleavage site just before the characteristic DINGGG residue (Figure 1B). The fact that a type II secretion system is present in the *psp *locus and that Psp has a signal peptide led us to predict that Psp is an extracellular protein secreted by the Hxc system. This hypothesis was experimentally tested and described below.

**Figure 2:**
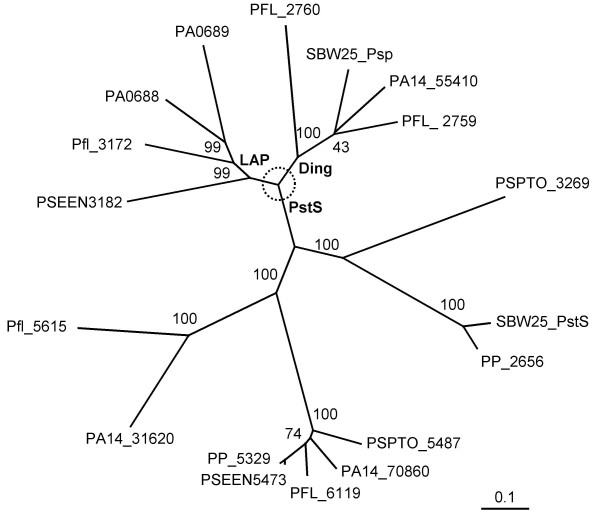
**The neighbor-joining tree showing phylogenetic relationships of putative phosphate-binding proteins from *Pseudomonas***. The deduced amino acid sequences are derived from the *Pseudomonas *genome databases: *P. aeruginosa *PA14 (PA14_55410, PA14_70860 and PA14_31620); *P. aeruginosa *PAO1 [PA0688 (LapA), PA0689 (LapB) and PA5369 (it is identical to PA14_70860 thus not shown in the tree); *P. putida *KT2440 (PP_5329, PP_2656); *P. fluorescens *Pf-5 (PFL_2759, PFL_2760 and PFL_6119); *P. fluorescens *Pf0-1 (Pfl_3172 and Pfl_5615); *P. syringae *pv. *tomato *DC3000 (PSPTO_3269 and PSPTO_5487); *P. entomophila *L48 (PSEEN3182 and PSEEN5473). Percentage bootstrap values obtained from 1000 trials are shown on branches. The scale bar refers to the number of substitutions per site.

Comparative genomic analysis of putative phosphate-binding proteins between *P. fluorescens *SBW25 and other genome-sequenced *Pseudomonas *showed that PstS is ubiquitous whereas the DING protein is only present in two other species or strains (*P. fluorescens *Pf-5 and *P. aeruginosa *PA14). *P. aeruginosa *PAO1 contains two DING-like proteins (LapA and LapB), of which the N-termini (-VTGGG) show partial similarity to the DINGGG residues. The *lapAB *genes were previously shown to encode low-molecular-weight alkaline phosphatases [14,17]. A phylogenetic tree based on the deduced amino acid sequences is shown in Figure 2. Clearly, the DING proteins form a separate group from the DING-like (LAP) and the PstS proteins, as already reported [8], but they are much more diverse than previously anticipated.

### The *psp *and *pst *operons are induced by P_i _limitation

To determine the conditions under which Psp is expressed, a transcriptional '*lacZ *fusion was constructed to *psp *and the resulting fusion plasmid (pUIC3-61) was integrated into the genome of SBW25 by a single event of homologous recombination. The resulting *psp*-'*lacZ *fusion strain (PBR829) was subjected to a β-galactosidase assay using cells grown in both phosphate-rich (PR) and phosphate-limited (PL) medium. Results (Table 1) showed that *psp *expression was elevated 3.85-fold at 4 hours and 64.72-fold at 26 hours, in cells grown in PL compared to cells grown in PR medium.

**Table 1:** Expression of the Psp and Pst genes in *P. fluorescens *SBW25

*lacZ *fusion	Medium	β-galactosidase (amol 4 MU/min/cell)^a^	
		
		4 hours	26 hours
*psp*-'*lacZ*	PR	0.22 ± 0.03	0.22 ± 0.07
	PL	0.83 ± 0.35 (3.85)	14.47 ± 3.53 (64.72)
*pstC*-'*lacZ*	PR	0.1 ± 0.01	0.16 ± 0.02
	PL	0.14 ± 0.02 (1.48)	0.58 ± 0.06 (3.61)

^a ^Fold of increase for cells grown in PL medium versus PR medium is shown in parenthesis.

Previous studies on transcription of *pst *genes from *P. aeruginosa *PAO1 and *E. coli *showed that the *pst *operon is expressed under P_i _limitation [7,18]. To test whether the predicted *pst *operon of SBW25 is induced by P_i _limitation, a *pstC*-'*lacZ *fusion strain (PBR830) was constructed and its expression was tested in a similar way as the *psp*-'*lacZ *fusion strain. Results (Table 1) showed that *pstC *expression was significantly higher for cells grown in PL medium compared with cells grown in PR medium. Compared to the levels of *psp *expression, the basic *pstC *expression and P_i _limitation-induced expression are very low (Table 1).

Next we carefully examined the promoter regions of *psp *and *pst *to search for putative Pho box sequences. As shown in Figure 1C, sequence motifs similar to the *E. coli *Pho box consensus were identified upstream of the translational start site of *pstS *and *psp*, suggesting that *psp *and *pst *operons are subjected to control by the conserved Pho regulon [7,19].

### Mutagenesis analysis of *psp *and *pstC*

Having demonstrated that *psp *and *pstC *are induced by P_i _limitation, we next asked whether they are required for bacteria to grow in laboratory medium with limited P_i _and in the plant environment. To do this, two in-frame deletion mutants PBR826 (Δ*psp*) and PBR827 (Δ*pstC*) were constructed by a standard procedure of allelic exchange. The two mutant strains showed similar growth curves with the wild-type when growing in complete medium (LB), minimal medium (M9) and phosphate media PR and PL (data not shown).

**Figure 3:**
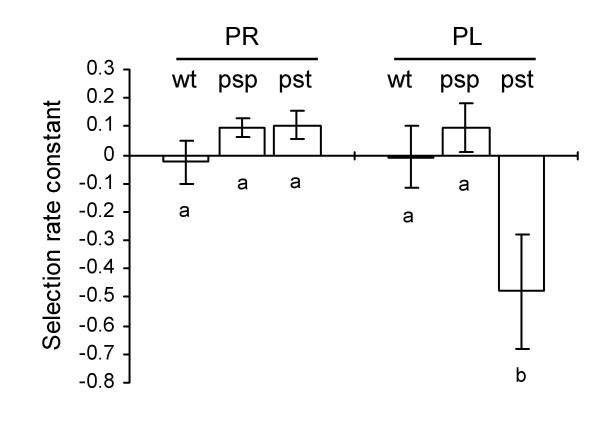
**Fitness of *P. fluorescens *SBW25 and derived mutants**. Fitness of SBW25 (wt) and mutants PBR826 (Δ*psp*, psp) and PBR827 (Δ*pstC*, pst) relative to SBW25-*lacZ *was measured with cells grown in P_i_-rich (PR) and P_i_-limited (PL) media. Data are means and standard errors of ~8 independent cultures. A fitness of zero indicates that the fitness of the mutant is identical to wild type (a negative value indicates a reduction in fitness relative to wild type). Bars identified by different letters are significantly different (*P *< 0.05) by Tukey's HSD.

To assess the growth more rigorously, we determined the competitive ability of each mutant relative to the wild type ancestor in two laboratory media (PR and PL). The mutant strain was mixed 1:1 with a *lacZ*-marked "wild type" strain (SBW25-*lacZ*) and the bacterial mixture was inoculated into the tested media. After growing in competition for ~20 generations in the laboratory media, the ratio of the mutant to the wild type competitor was determined on LB plus X-gal agar plates. Results are shown in Figure 3. Growth of PBR827 (Δ*pstC*) was comparable to the wild-type in PR medium but was significantly impaired in PL medium. No fitness effect was observed with PBR826 (Δ*psp*).

The competitive ability of each mutant (Δ*psp *and Δ*pstC*) relative to the *lacZ*-marked "wild type" strain was also determined during a course of colonization of sugar beet seedlings. Each mutant strain was inoculated, with the wild type in a 1:1 ratio, onto sugar beet seeds and recovered in two weeks from the shoots and rhizosphere on M9 plates supplemented with X-gal. Results showed that the fitness of PBR826 (Δ*psp*) and PBR827 (Δ*pstC*) was not significantly impaired in either the shoots or the rhizosphere (data not shown).

### Psp is secreted by the Hxc system

Armed with knowledge that *psp *is expressed in P_i _limited medium, we next asked whether Psp is an extracellular protein and furthermore whether it is secreted via the Hxc system. An immunodiffusion experiment was performed between anti-DING antiserum and concentrated supernatants of SBW25 (wild-type) cultures in PL and also in PR and LB broths (as controls). Results showed that a precipitin line was formed only between anti-DING serum and PL supernatant (data not shown). From this experiment we conclude that Psp is an exoprotein, and its expression is induced by P_i _limitation, which is consistent with the β-galactosidase activity data described above and is further confirmed by the Western blotting analysis described below.

**Figure 4:**
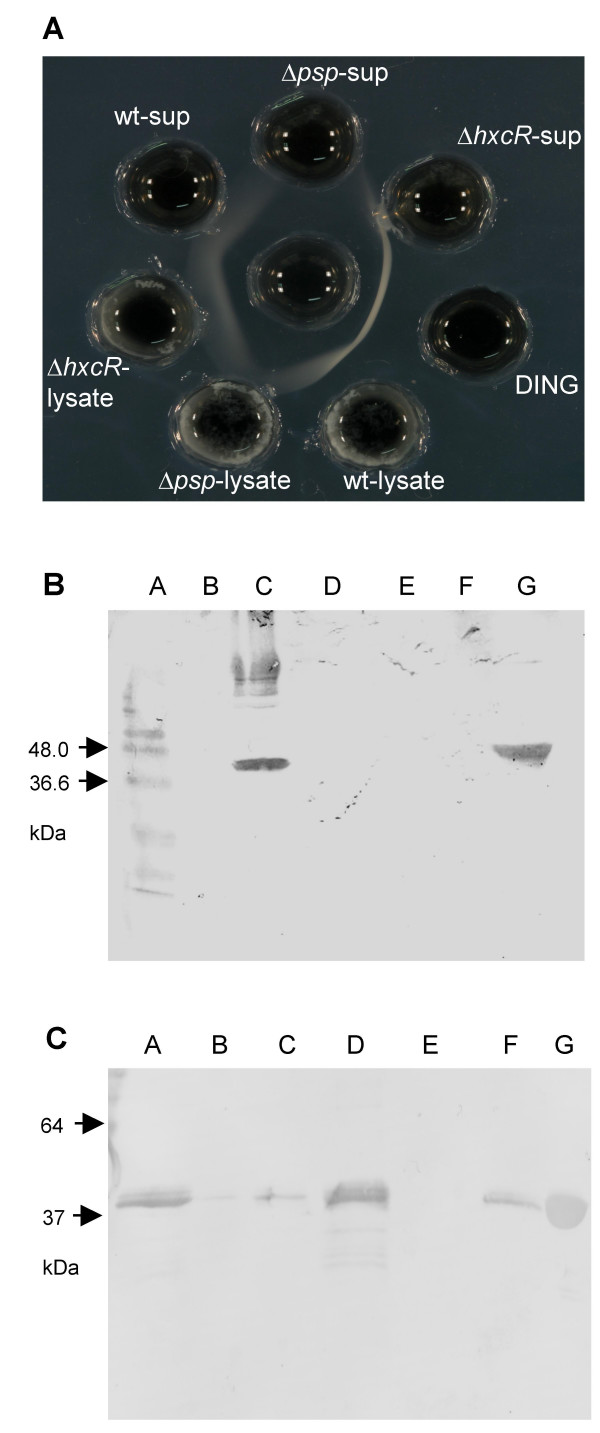
**Immunochemical analysis of Psp in *P. fluorescens *SBW25 and derived mutants**. **(A) **Double immunodiffusion of cell supernatants and lysates. The central well in a 1.5% agarose plate contained rabbit anti-DING (Psp from SBW25) antiserum. The peripheral wells contained (clockwise, from the top) PBR826 (Δ*psp*) supernatant (Δ*psp*-sup), PBR828 (Δ*hxcR*) supernatant (Δ*hxcR*-sup), Psp standard (DING), SBW25 lysate (wt-lysate), PBR826 (Δ*psp*) lysate (Δ*psp*-lysate), PBR828 (Δ*hxcR*) lysate (Δ*hxcR*-lysate), SBW25 supernatant (wt-sup). **(B) **Western blotting of supernatants for SBW25 grown in different media. Concentrated cell supernatants were separated on a 12% SDS-PAGE gel, and Psp was detected with anti-human DING antiserum. Lane A, protein ladder; B, PBR826 (Δ*psp*) in LB broth (negative control); C, SBW25 in PL broth; D, E, SBW25 in PR broth; F, SBW25 in LB broth; G, recombinant Psp (positive control). **(C) **Western blotting of cell supernatants for SBW25 mutants grown on PL medium. Concentrated cell fractions were separated on a 12% SDS-PAGE gel, and Psp was detected with anti-Psp antiserum. Lane A and D, wild-type; B and E, PBR826 (Δ*psp*); C and F, PBR828 (Δ*hxcR*); G, Psp standard.

To test whether Psp secretion is dependent on the Hxc system, an in-frame *hxcR *(*pflu2424*) deletion mutant was constructed by allelic exchange. *hxcR *encodes an essential component of the type II secretion system (ATPase), which provides energy for substrate translocation. Concentrated supernatants and cell lysates of the mutant strain PBR828 (Δ*hxcR*) grown in PL medium were subjected to immunodiffusion analysis with an anti-DING antibody. Wild-type SBW25 and PBR826 (Δ*psp*) were included and tested in parallel as the positive and negative control, respectively. Figure 4A shows that Psp is present in both supernatant and lysate of wild-type cells, but is not present in the lysate or supernatant from PBR826 (Δ*psp*). In PBR828 (Δ*hxcR*), Psp is detectable at normal concentrations in the lysate, but is present at much lower concentrations in the supernatant, indicating that secretion is significantly reduced by the Δ*hxcR *mutation.

This result was confirmed by Western blot analysis of the same bacterial supernatants: only in PL medium was a 40 kDa protein detectable with anti-DING antibodies (Figure 4B). Figure 4C showed that DING was detected as a strong band in the supernatant of the wild-type culture, but was totally absent in mutant PBR826 (Δ*psp*), and greatly reduced (< 15%) in the PBR828 (Δ*hxcR*) supernatant. This Western blot result indicates that the Psp secretion is predominantly via the Hxc system.

### HxcR is not induced by P_i _limitation but shows elevated level of expression *in planta*

The *in vivo *expression technology (IVET) has been employed to identify SBW25 genes showing elevated levels of expression on the plant surfaces [20,21]. These plant inducible genes are of particular interest because they are predicted to play a role in the maximization of ecological performance *in planta *– a prediction confirmed for a locus encoding an acetylated cellulose polymer [20] and *cueA*, a gene encoding the copper-transporting P-type ATPase [22].

Central to this technique is a *dapB *deletion mutant strain (SBW25Δ*dapB*), which is unable to grow in the absence of an exogenous source of DAP (diaminopimelic acid) and lysine and, as a result, is unable to colonize sugar beet seedlings [20]. By fusing random fragments of the SBW25 genome to a promoterless copy of *dapB *it is possible to identify plant-inducible loci. Arising from a genome-wide screen for plant-inducible loci from SBW25 was strain PIL082, which contains a fusion between *hxcR *and '*dapB *(Figure 1B) suggesting that the *hxc *locus is plant-induced.

Experimental evidence described above showed that *psp *is induced by P_i _limitation, but the precise location of the *psp *promoter was unclear. *hxcR *and *psp *are organized in the same orientation. Thus it is possible that *psp *is cotranscribed with *hxcRS *and induced also in the plant environment. To test this hypothesis, an IVET fusion (*psp*-'*dapB*) was constructed by cloning an 800 nt DNA fragment from *hxcS *to *psp *into pIVETD, and integrated into the genome by homogenous recombination. The resultant IVET fusion (PIL082-1) was subjected to a plant colonization assay, together with the *hxcR *IVET fusion strain (PIL082). About 1000 cells were inoculated onto each sugar beet seed and bacteria were recovered from the shoots and rhizosphere, two weeks after plant sowing. Results showed that the *psp *IVET fusion strain was not detected (under the detection limit of 30 cells per shoot, 300 cells per rhizosphere), whereas the *hxcR *fusion strains reach a cell density of 6.7 ± 3.4 × 10^2 ^cells per shoot and 5.46 ± 2.30 × 10^4 ^per rhizosphere. Both PIL082 and PIL082-1 are unable to grow in minimal medium (M9) in the absence of lysine and DAP. The ability of PIL082 to grow on sugar beet seedlings but not on M9 medium confirmed that the promoter driving *hxcR *expression is active in the plant environment. The inability of PIL082-1 to grow *in planta *indicates that *psp *is not induced in the plant environment.

To test whether *hxcR *is induced by P_i _limitation, expression of PIL082 fusion was examined by measuring the β-galactosidase activities in cells grown in PR and PL media (the IVET vector pIVETD contains a second marker gene '*lacZ *located downstream of '*dapB*) [20]. Results showed that *hxcR *expression was not elevated in cells grown in PL medium (0.84 ± 0.06 amol 4 MU/min/cell) compared to cells grown in PR medium (0.97 ± 0.02 amol 4 MU/min/cell). Taken together, the expression data (both *in vitro *and *in vivo*) indicate that *hxcR *and *psp *are transcribed by different promoters. Given that *hxcR *and *hxcS *are organized in a contiguous manner, the *psp *promoter is likely to be located in the intergenic region of *hxcS *and *psp *(Figure 1B). This is consistent with the tentative identification of a putative Pho box in this region (Figure 1C).

## Discussion

Phosphate is an essential but often limiting nutrient. Multiple P_i _transport systems have evolved in bacteria to facilitate efficient uptake of P_i _from the environment. In *E. coli*, a constitutively-expressed, low-affinity transporter (Pit system) is used in cells grown in environments with excess P_i_. Under conditions of P_i _limitation, a high-affinity transport system (Pst) is induced and operates for P_i _scavenging [7]. In this report, we identified by *in silico *analysis genes encoding the Pst transporter system in the SBW25 genome, whose function we experimentally tested. The Pst system is an ABC-type transporter that takes up P_i _from the periplasmic space of Gram negative bacteria. Psp, a protein secreted outside of the cell, may act to scavenge P_i _to the cell surface, where it can be subsequently taken up by the Pst system. Therefore, the Psp and Pst systems are very likely involved in phosphate acquisition in a cooperative manner. Although *psp *is induced by limiting phosphate concentrations, its absence does not limit bacterial competitive growth under laboratory conditions, unlike *pstC*. The DING-like LAP proteins may hydrolyze environmental organophosphates, following secretion, which is also consistent with a phosphate-scavenging role. It is noteworthy that PstS sequences which are duplicated in some *Pseudomonas *genomes may be significantly different, implying that there may also be functional variance within this sub-family of phosphate-binding proteins.

Bacteria living in complex environments, e.g. plants and animals, utilize the type II secretion pathway for extracellular release of particular proteins, such as toxins and hydrolytic enzymes [15]. Two functionally independent type II secretion systems (Xcp and Hxc) have been described in *P. aeruginosa *[14]. Interestingly, in addition to the Xcp and Hxc homologues, the *P. fluorescens *SBW25 genome harbors an additional copy of the Type II gene cluster (Rainey lab, unpublished). Here we showed that the DING protein secretion was greatly reduced in the HxcR mutant PBR828 (Δ*hxcR*), suggesting that there may be some functional redundancy amongst the three type II secretion systems.

Psp is regulated by P_i _limitation but its expression is not elevated in the plant environment, indicating that P_i _is not depleted in our plant growth system. On the contrary, *hxcR *is not induced by P_i _limitation but shows elevated levels of expression in sugar beet seedlings. While the result implicates important roles of the Hxc secretion system in bacterium-plant interaction, like most plant-inducible genes [20,21], the signals that induce *hxcR *expression *in planta *are currently unknown.

## Conclusion

Proteins belonging to the DING family have very poorly defined physiological roles. Functional investigation of eukaryotic DING proteins is hindered by the unavailability of full amino acid sequences in most cases and by the absence of full gene sequences in the eukaryotic genome and EST databases. Insight into the function and gene regulation of the prokaryotic members of DING protein family may provide valuable cues as to the functions of the DING proteins in general. A previous study showed that Psp from *P. fluorescens *SBW25 is capable of binding P_i _*in vitro *[9]. In this report we demonstrated in *P. fluorescens *SBW25 that Psp is expressed under P_i _limitation and is an exoprotein, secretion of which is mainly dependent on the alternative type II secretion system (Hxc). Interestingly, expression of the Hxc system is elevated on plant surfaces, which is first reported here. Moreover, the Pst P_i _transporter system was predicted in the SBW25 genome and its function was experimentally characterized in parallel to Psp. Results show that Pst is induced by phosphate limitation and it is required for bacterial competitive growth in phosphate limiting medium. Taken together, the data implicate functional roles of Psp in bacterial phosphate metabolism. Psp and Pst could form an efficient phosphate scavenging system, which contributes to physiological adaptation to phosphate limited environments.

## Methods

### Bacterial strains, plasmids and growth conditions

The ancestral strain of *P. fluorescens *SBW25 is a plasmid-free, non-pathogenic bacterium belonging to the rRNA group I of fluorescent *Pseudomonas *isolated from sugar beet phytosphere [10]. *Escherichia coli *DH5αλ*pir *[23] was used as a recipient strain for gene cloning and then a donor for conjugative transfer into *Pseudomonas *cells. A summary of other bacterial strains and plasmids used in this study is provided in Table 2. *P. fluorescens *and *E. coli *strains were routinely grown in Luria-Bertani (LB) medium [24] at 28°C and 37°C, respectively. In P_i _limitation studies, *P. fluorescens *strains were grown in a defined citrate medium [25] that contains citric acid (4.0 g/l), (NH_4_)_2_SO_4 _(1.0 g/l) and MgSO_4_·7H_2_O (0.2 g/l). Additionally, Na_2_HPO_4 _and KH_2_PO_4 _were added at the concentration of 6.0 g/l and 3.0 g/l, respectively in phosphate rich medium (PR), whereas in phosphate-limited (PL) medium they are added at 2.4 mg/l and 1.2 mg/l, respectively. IVET fusion strains of SBW25Δ*dapB *were cultivated in minimal M9 medium [24] supplemented with diaminopimelate (DAP) and lysine at the concentration of 800 μg/ml and 60 μg/ml, respectively. A *Pseudomonas *selective CFC (Cetrimide, Fucidin, and Cephalosporin) from Oxoid (Hampshire, UK) was supplemented in the LB agar plates to select for *P. fluorescens *recovered from plant. When necessary, antibiotics were added to the following concentrations (μg/ml): tetracycline (Tc), 15; kanamycin (Km), 50; Streptomycin (Sm), 100; nitrofurantoin (NF), 100. Nutritional supplements of diaminopimelate (DAP) and lysine were added at the final concentration of 800 μg/ml and 60 μg/ml, respectively.

**Table 2:** Bacterial strains, plasmids and oligonucleotide primers used in this work

Strain, plasmid or primer	Relevant properties	Source/reference
*P. fluorescens*		
SBW25	Wild type strain isolated from sugar beet	[10]
SBW25-Sm	Spontaneous Sm^r ^derivative of SBW25	[20]
SBW25Δ*dapB*	DAP/lysine auxotroph of SBW25	[20]
PIL082	The *hxcR*-'*dapB'lacZ *IVET fusion strain of SBW25Δ*dapB*, Tc^r^	This work
PIL082-1	Δ*dapB *DUP(*hxcS*-*psp*)::pIVETD, the *psp*-'*dapB'lacZ *IVET fusion strain, Tc^r^	This work
SBW25-*lacZ*	SBW25 marked with '*lacZ *in a phage locus	[28]
PBR826	Δ*psp *or Δ*pflu2427*, SBW25 with a nonpolar deletion of *psp*, Tc^s^	This work
PBR827	Δ*pstC *or *Δpflu3317*, SBW25 with a nonpolar deletion of *pstC*, Tc^s^	This work
PBR828	Δ*hxcR *or Δ*pflu2424*, SBW25 with a nonpolar deletion of *hxcR*, Tc^s^	This work
PBR829	DUP(*hxcS*-*psp*)::pUIC3, the *psp*-'*lacZ *fusion strain of SBW25, Tc^r^	This work
PBR830	DUP(*pstS*-*pstC*)::pUIC3, the *pstC*-'*lacZ *fusion strain of SBW25, Tc^r^	This work
		
Plasmid		
pRK2013	Helper plasmid, Tra^+^, Km^r^	[34]
pUIC3	Integration vector with promoterless '*lacZ*, *ori*R6K, Mob^+^, Tc^r^	[21]
pIVETD	DAP-based IVET vector, pUIC3 with promoterless '*dapB*'*lacZ*, *ori*R6K, Tc^r^	[20]
pIVETD-13	pIVETD containing 0.8 kb *psp *fragment fused to '*dapB*'*lacZ*, *ori*R6K, Tc^r^	This work
pUIC3-61	pUIC3 carrying *psp*-'*lacZ *fusion, *ori*R6K, Tc^r^	This work
pUIC3-62	pUIC3 carrying *pstC*-'*lacZ *fusion, *ori*R6K, Tc^r^	This work
pUIC3-41	pUIC3 containing 1.6 kb *psp *deletion fragment, *ori*R6K, Tc^r^	This work
pUIC3-63	pUIC3 containing 1.6 kb *pstC *deletion fragment, *ori*R6K, Tc^r^	This work
pUIC3-64	pUIC3 containing 1.9 kb *hxcR *deletion fragment, *ori*R6K, Tc^r^	This work
Primer^a^		
psp-1	GAAGATCTGTTGCGTGCCCTGGAAG	
psp-2	cagcatgcggatccgttgacggaAGCGAGGGTCATGGATACCG	
psp-3	tccgtcaacggatccgcatgctgCGGCAACACCAACGTCTGC	
psp-4	GAAGATCTGCTGCTGGAAATCCACGGT	
hxcR-1	GAGATCTGCGGTAATGGCCACCTATG	
hxcR-2	cagcatgcggatccgttgacggaCGCTGCACCTCGCTGATCGA	
hxcR-3	tccgtcaacggatccgcatgctgTCGACGACGATGTGCGCAGC	
hxcR-4	GAGATCTATGCCAATTCAAACGCGCCA	
pstC-1	GAGATCTTCACCAAGCACCTGGCGG	
pstC-2	cagcatgcggatccgttgacggaAGCCGTGCTTTTCCATGCCT	
pstC-3	tccgtcaacggatccgcatgctgTCGCTGTTTGCACCGGCCAAC	
pstC-4	GAGATCTGCCGGTGGGCAAGACGATTTTC	

^a ^Restriction sites incorporated into the primers are underlined. Complementary sequence designed for the SOE-PCR is indicated by small letters.

### DNA manipulations

General DNA recombination techniques were used according to standard protocols [24]. Plasmids and DNA fragments from agarose gels were extracted and purified using kits from Qiagen (Biolab Ltd., Auckland, NZ). DNA restriction and modification enzymes and T4 DNA ligase were purchased from Roche (Auckland, NZ) and used as recommended by the manufacturer. *P. fluorescens *SBW25 genes were amplified from genomic DNA by polymerase chain reactions (PCR) using *Taq *DNA polymerase from Invitrogen (Auckland, NZ). DNA was sequenced using the BigDye Terminator Sequencing kit (Applied Biosystems, Auckland, NZ) on an automated DNA Sequencer, model 310 (Perkin Elmer).

### Construction of *lacZ *fusions and assays for β-galactosidase activity

To construct *lacZ *fusions to *psp *and *pstC*, DNA fragments (~800 bp) were amplified from *P. fluorescens *SBW25 by using primer pairs "psp-1/-2" and "pstC-1/-2", respectively. The PCR products were first cloned into pCR8/GW/TOPO (Invitrogen, Auckland, NZ) and sequence identity was confirmed by DNA sequencing. The DNA was retrieved by *Bgl*II and *Bam*HI digestion and cloned into the *Bgl*II site of pUIC3 (Mob^+^, Tra^-^). Gene orientation was then determined by restriction analysis of *Bgl*II and *Eco*RI. The resulting plasmid (pUIC3-61 or pUIC3-62) was mobilized into *P. fluorescens *SBW25 by a general procedure of plasmid conjugation with the help of pRK2013 (Mob^+^, Tra^+^). Transconjugants were selected on LB plates supplemented with nitrofurantoin (to counter-select *E. coli*) and Tc. The recombinant plasmid integrated into the genome by a single event of insertion-duplication. Correct integration was confirmed by PCR.

β-galactosidase activities were assayed by using 4 MUG (4-methylumbelliferyl-β-D-galactoside) as the enzymatic substrate. The product (7-hydroxy-4-methylcoumarin, 4 MU) was detected using a Hoefer DyNA Quant 200 fluorometer (Pharmacia Biotech, Auckland, NZ) with an emission and excitation wavelength of 460 nM and 365 nM, respectively. Cell density was determined by measuring the absorbance of the culture at 600 nm. The enzyme activity was expressed as "amol 4 MU/min/cell".

### Site-directed mutagenesis of *P. fluorescens *SBW25 and fitness assays

The *psp*, *pstC *and *hxcR *deletion mutants were constructed by a previously described protocol of SOE-PCR (splicing by overlapping extension using the polymerase chain reaction) [26] in conjunction with a two-step allelic exchange strategy. Two DNA fragments flanking the deleted region of a gene were amplified by two primer pairs, e.g. psp-1/psp-2 and psp-3/psp-4 in the case of *psp *deletion. The two DNA fragments were then joined together by PCR reaction using primers psp-1 and psp-4 (this was possible because of the complementary sequences incorporated into primers psp-2 and psp-3). The resulting ~1.6 kb DNA fragment was cloned into pCR8/GW/TOPO and sequenced to ensure that it was error-free. The DNA fragment was retrieved by *Bgl*II then cloned into the delivery vector pUIC3 to generate pUIC3-41 (for Δ*psp*), pUIC3-63 (for Δ*pstC*) and pUIC3-64 (for Δ*hxcR*).

To delete *psp *(or *pstC *or *hxcR*) from the genome of SBW25, pUIC3-41 (or pUIC3-63 or pUIC3-64) was mobilized into SBW25 by conjugation with the help of pRK2013 (Tra^+^). Integration by homologous recombination was selected on LB plates supplemented with nitrofurantoin, tetracycline and X-gal. To select for allelic exchange mutants, purified single blue-colored transconjugants were subjected to cycloserine enrichment as previously described [27]. Allelic exchange mutants (white-colored and Tc-sensitive) were examined by PCR to distinguish the mutants from wild type.

Fitness of the mutant strains in laboratory media and *in planta *was assessed by direct competition with a *lacZ*-marked "wild-type" strain of *P. fluorescens *SBW25 (SBW25-*lacZ*) as previously described [28]. The mutant and the wild-type competitor were counted on LB agar plates supplemented with X-gal. Population densities (*N*_*i*_) determined at time *t *= 0 and at *t *= *T *were used to calculated the Malthusian parameter [29], which is the average rate of increase and was calculated for both competitors: *m*_*i *_= ln [*N*_*i*_(*T*)/*N*_*i*_(0)]. Relative fitness is expressed here as the selection rate constant (SRC): *r*_*ij *_= *m*_*i *_- *m*_*j*_, resulting in a fitness of zero when competing organisms are equally fit.

### *In vivo *expression technology analysis and plant experiments

Isolation process of the PIL082 fusion was previously described [20]. To construct the *psp *IVET fusion, the ~800 bp DNA fragment used for the construction of *psp*-*lacZ *fusion (pUIC3-61) was cloned into the *Bgl*II site of the DAP-based IVET vector pIVETD to generate pIVETD-13, which has *psp *fused to the promoterless '*dapB *reporter. PIVETD-13 was then integrated into the *psp* locus of SBW25 by conjugation with the help of pRK2013. The obtained *psp *IVET fusion strain was named PIL082-1.

Ability of the IVET strains to grow *in planta *was assessed by a competitive colonization assay in sugar beet seedlings [22]. The fusion strain was mixed 1:1 with a competitor strain (SBW25-Sm) and inoculated onto coated sugar beet (*Beta vulgaris *var. *Amethyst*) seeds, which were germinated and cultivated in 15 ml plastic tubes filled with non-sterile vermiculite. About 1000 cells were inoculated and two weeks after plant sowing bacteria from the shoot and rhizosphere (roots with attached vermiculite) were counted on LB plates supplemented with lysine, DAP, CFC and X-gal. The competitor strain SBW25-Sm was counted in LB plates with CFC and streptomycin and present in all treatments at the similar levels (~10^4 ^per shoot and ~10^6 ^per rhizosphere).

### Immunochemical analysis

Two antisera were used in these experiments. A rabbit antiserum to a conjugated, synthetic peptide corresponding to the N-terminus of the human DING protein, was prepared and used as previously described [3]. The second antiserum (anti-Psp) was made in NZ White rabbits, with recombinant DING protein from *P. fluorescens *SBW25 [9] as the antigen, using standard methods. Double immunodiffusion was carried out in 1.5% (w/v) agarose gels. Western blotting was carried out as previously described [3], using 12% precast SDS-PAGE gels (Bio-Rad, Auckland, NZ). *The wild-type *SBW25 and mutant strains Δ*psp *and Δ*hxcR *were grown to the same cell density in 5 ml cultures. Supernatants were freeze-dried, and resuspended in 0.1 ml 30 mM Tris-HCl buffer (pH 7.5). Cell pellets were similarly resuspended, sonicated and re-centrifuged. For immunodiffusion, 0.025 ml samples were used, and for electrophoresis, 0.015 ml, in each case. Triplicate cultures were analyzed, and a typical result is shown in each case.

### Computational analysis

PstS was identified by BLAST (basic local alignment search tool) search of the complete SBW25 genome sequence using the deduced amino acid sequence of *psp *(*pflu2427*). The SBW25 Psp and PstS sequences were then used in BLAST searches for homologues in other *Pseudomonas *species or strains deposited in the *Pseudomonas *genome database v2 [30]. Amino acid sequences were aligned using ClustalX program [31] and a phylogenetic tree constructed using the neighbor-joining method [32]. The tree was displayed in TreeView [33].

## Abbreviations

P_i_, inorganic phosphate; IVET, *In vivo *expression technique; DAP, diaminopimelic acid; SRC, selection rate constant; EST, expressed sequence tag; P_i_, inorganic phosphate; LB, Luria-Bertani; PR, phosphate rich; PL, phosphate limited; CFC, cetrimide, fucidin, and cephalosporin; NF, nitrofurantoin; Tc, tetracycline; Km, kanamycin; Sm, streptomycin. PCR, polymerase chain reactions. 4 MU, 7-hydroxy-4-methylcoumarin.

## Authors' contributions

XXZ, KS and PBR designed the experiments and interpreted the results. XXZ performed the genetic manipulations, enzymatic assays and plant experiments and drafted the manuscript. KS conducted the immunochemical experiments, and contributed to the manuscript. RM was involved in the genetic and immunochemical studies. All authors read and approved the final manuscript.
